# Colloid cyst of the third ventricle, hypothalamus, and heart: a dangerous link for sudden death

**DOI:** 10.1186/1746-1596-7-144

**Published:** 2012-10-18

**Authors:** Emanuela Turillazzi, Stefania Bello, Margherita Neri, Irene Riezzo, Vittorio Fineschi

**Affiliations:** 1 Department of Forensic Pathology, University of Foggia, Foggia, Italy; 2 Department of Forensic Pathology, University of Foggia Ospedale Colonnello D’Avanzo, Via degli Aviatori 1, 71100, Foggia, Italy

**Keywords:** Colloid cyst of the third ventricle, Hypothalamus stimulation, Contraction band necrosis, Sudden death

## Abstract

**Abstract:**

Colloid cysts are rare congenital, intracranial neoplasms, commonly located in the third ventricle. Colloid cysts are endodermal congenital malformations. The cysts commonly range in size from 1–2 cm in diameter, although large cysts >3 cm in size have been reported. The components of the cyst include an outer fibrous capsule over an inner epithelium. The epithelium is usually a single layer of mucin-producing or ciliated cells. Such cysts contain mucoid and gelatinous material, which is positive for both Periodic acid Schiff (PAS) and mucicarmen staining. Although colloid cysts usually represent histopathologically benign neoplasms, they can result in sudden, unexpected and potentially lethal complications. The mechanism(s) of death is still a controversial subject and several mechanisms have been postulated to explain the sudden onset of severe symptoms and of fatal rapid deterioration in patients with colloid cysts. In this case, macroscopic and histological findings addressed the diagnosis of colloid cyst of the third ventricle with diffuse myocardial injury (coagulative myocytolysis or contraction band necrosis, CBN) and led us to conclude that acute cardiac arrest due to hypothalamus stimulation in the context of colloid cyst of the third ventricle was the cause of death. As the hypothalamic structures which are involved in neuroendocrine and autonomic regulation playing a key role in cardiovascular control are located close to the walls of the third ventricle which is the most frequent anatomical site of colloid cyst, this may suggest that reflex cardiac effects due to the compression of the hypothalamic cardiovascular regulatory centers by the cyst explain the sudden death in patients harboring a colloid cyst when signs of hydrocephalus or brain herniation are lacking.

**Virtual slides:**

The virtual slide(s) for this article can be found here: http://www.diagnosticpathology.diagnomx.eu/vs/4915842848034158

## Background

Colloid cysts are rare congenital, intracranial neoplasms, commonly located in the third ventricle, accounting for 0.2-2% of all intracranial and approximately for 15–20% of intraventricular neoplasms, respectively. Colloid cysts are slow growing and the initial onset of symptoms is usually between 20 and 50 years of age, although they have been reported also in younger patients [1]. Diagnosis during childhood is unusual and only 1-2% of all reported cases occurred during the patients’ first decade [2].Although colloid cysts usually represent histopathologically benign neoplasms, they can result in sudden, unexpected and potentially lethal complications. The mechanism(s) of death is still a controversial subject and several mechanisms have been postulated to explain the sudden onset of severe symptoms and of fatal rapid deterioration in patients with colloid cysts [3].

Here we present the death of a previously healthy young boy harboring a colloid cyst of the third ventricle. We investigated the possible mechanism of death through a careful histological examination of heart specimens in addition to gross and histological examination of the brain.

## Case presentation

A 10 year – old boy at 8.00 p.m. complained a severe headache accompanied by vomiting attacks. An emergency physician visited him and advised him to rest for some hours. He lost consciousness in the early morning and was transported to the local hospital, where he was pronounced dead upon arrival. The medical history taken from the parents showed no significant signs and symptoms except for repeated mild attacks of leg weakness. Familial history was negative both for cardiovascular and neurological diseases and sudden death.

**Figure 1:**
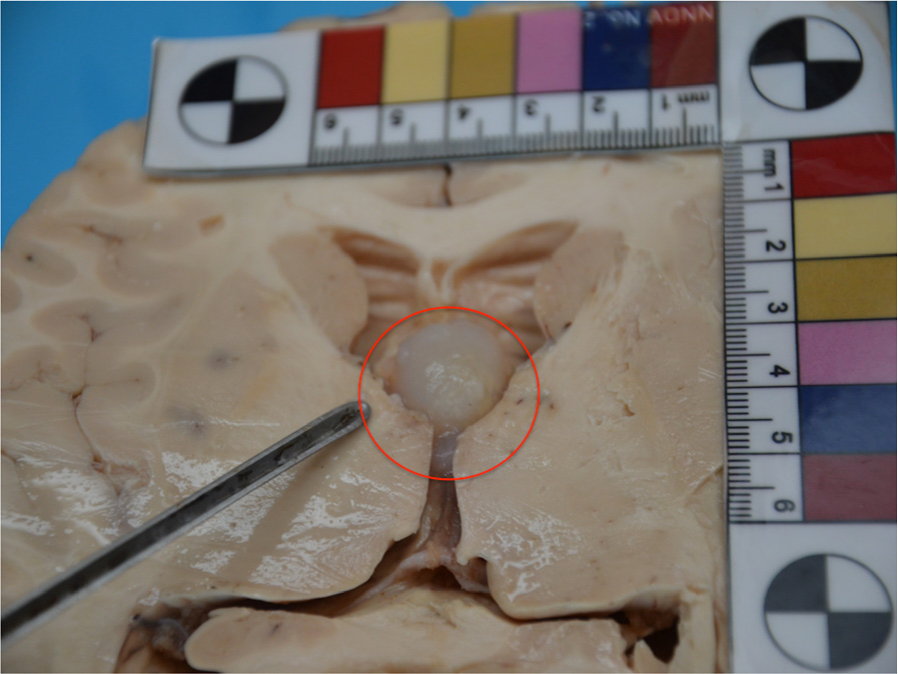
Brain section according horizontal plan: a 2cm pale grayish-white cyst partially filling and distending the third ventricle.

**Figure 2:**
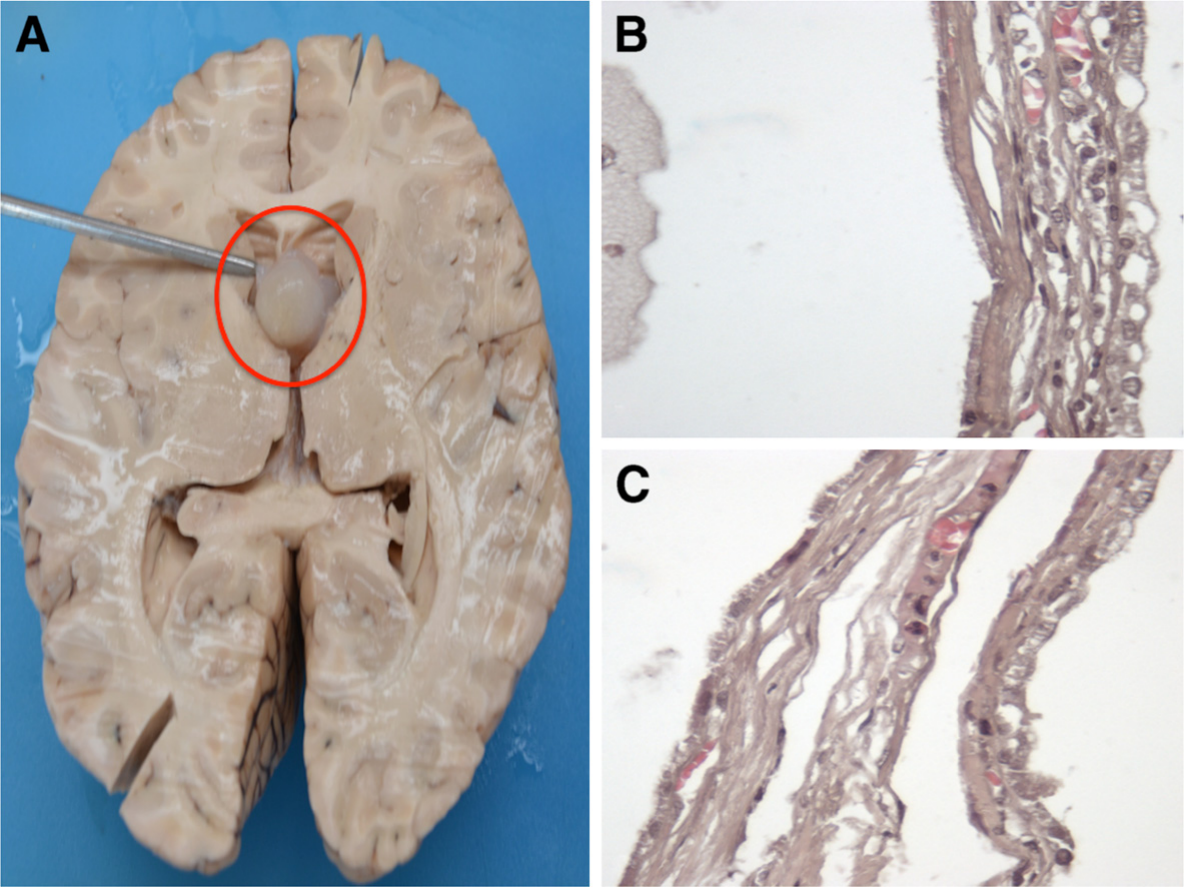
**(A) The cyst was wedged between the splayed columns of the fornix non obstructing the left foramen of Monro.** (**B**-**C**) The cyst was lined by a single layer of cuboidal, partially flattened, non – ciliated and ciliated epithelial cells.

At autopsy, gross examination showed an enlarged, edematous, symmetrical brain weighing 1650g. Marked brain swelling with gyral flattening, and sulcal narrowing were also detected. Both cerebellar tonsils were grooved. The cerebral ventricles, containing clear and colorless cerebral spinal fluid (CSF), were mildly widened. Unci were normal and the midbrain was free of haemorragic lesions. Other organs were unremarkable at gross observation. Due to the severe cerebral oedema, that would cause the death of the young boy, once removed, the brain was fixed in formalin. The successive gross examination of the 2 – weeks formalin fixed brain was performed by sectioning the specimen in the horizontal plan. Sections were notable for a 2cm pale grayish-white cyst partially filling and distending the third ventricle (Figure 1), which was wedged between the splayed columns of the fornix non obstructing the left foramen of Monro (Figure 2A). The lateral ventricles were mildly dilated. Sectioning of the cyst showed a viscid substance hardened after formalin fixation and a thin fibrouse capsule.

**Figure 3:**
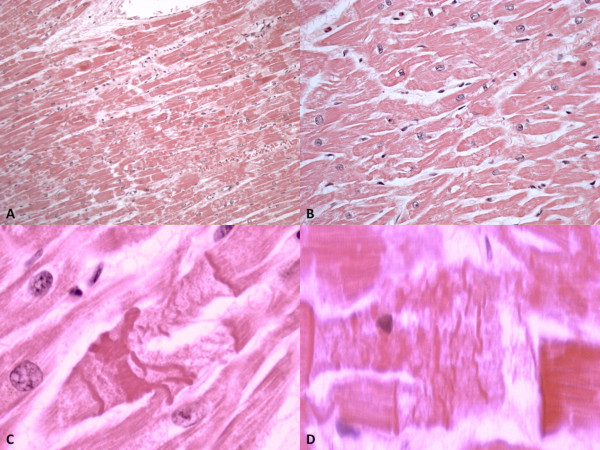
**Contraction band necrosis.** (**A**-**B**) Pancellular lesion with fragmentation of hypercontracted myofibrils and band formation of hypercontracted or coagulated sarcomeres (A H&E × 40, B H&E x 80). (**C**-**D**) Paradiscal lesion formed by about 15 hypercontracted sarcomeres without myofibrillar rhexis. Note the absence of edema, haemorrhage and myofibre vacuolisation (C H&E × 200, D H&E x 250).

Specimens from all organs were taken and stained with haematoxilyn and eosin (H&E). In addition, cyst’s samples were stained with periodic acid-Schiff (PAS) stain. Microscopic examination revealed a cystic lesion, filled of a strongly PAS positive amorphous content and lined by a single layer of cuboidal, partially flattened, non – ciliated and ciliated epithelial cells, resting on a thin collagenous membrane (Figure 2B-C). Histological examination of the brain revealed a severe edema. The pathological myocardial picture included two patterns of lesion. One corresponds to fragmentation of the whole myocell (pancellular lesion) in pathological band with intense hyperosinophilia of the hypercontracted myocardial cells, extremely short sarcomeres, highly thickened Z lines, and rexis of the myofibrillar apparatus into cross-fibre, anomalous and irregular (Figure 3A-B). Pathological bands were formed by segments of hypercontracted and coagulated sarcomeres. The second pattern associated to the previous one was represented by a unique band of 10–20 hypercontracted sarcomeres close to the intercalated disc with a typical aspect of paradiscal lesion (Figure 3C-D). In this case the band assumes a dark, dense ultrastructural aspect with very thin Z lines and myofibrils and mitochondria squeezed in the normal portion of the myocyte. Aspects of myocardial fibrosis were also detected. Histological examination of other organs was unremarkable.

Toxicological analyses were negative.

## Conclusions

In this case, macroscopic and histological findings addressed the diagnosis of colloid cyst of the third ventricle with diffuse myocardial injury (coagulative myocytolysis or contraction band necrosis, CBN) and led us to conclude that acute cardiac arrest due to hypothalamus stimulation in the context of colloid cyst of the third ventricle was the cause of death.

We observed two patterns of myocardial lesions. One corresponded to fragmentation of the whole myocell (pancellular lesion), which ranges from early breakdown in pathological bands to a total granular disruption (myofibrillar degeneration). The second pattern, associated with the previous one, is characterized by a unique band of 10 to 20 hyper-contracted sarcomeres close to the intercalated disc (paradiscal lesion). These lesions are typical of catecholamine myotoxicity and may express a sympathetic overstimulation.

As previously reported, in many conditions in which a sympathetic overtone is likely to be present, CBN is observed as a histological hallmark of an adrenergic storm [4]. Such myocardial pathological pattern is also described in many brain diseases. In fact there is a strong evidence that overactivity of the sympathetic limb of the autonomic nervous system is the common phenomenon that links the cardiac alterations seen in neurological fatal events. These effects on the heart may contribute in a major way to the mortality rates of many primarily neurological conditions such as subarachnoid hemorrhage, cerebral infarction, status epilepticus, and head trauma, acute intracranial hypertension [5], and may also be important in the pathogenesis of sudden death in the context of intraventricular colloid cyst. Neurogenic cardiac stunning (due to hyperacute, intense, neuronal sympathetic activation) may represent the pathological cardiac event occurring in response to sudden increase in intracranial cerebral pressure (ICP) in third ventricle colloid cyst context [6]. The cyst can fill the ventricle or obstruct the flow of CSF and lead to prominent hydrocephalus. Acute ventricular hydrocephalus with intracranial hypertension and brain herniation can result in cerebral compression, medullary (respiratory) compromise and death [7-10]. Sudden death has been also related to acute cyst swelling due to spontaneous intralesional bleeding that may cause acute obstructive hydrocephalus and intracranial hypertension due to rapid enlargement of the lesion itself [11-13].

**Figure 4:**
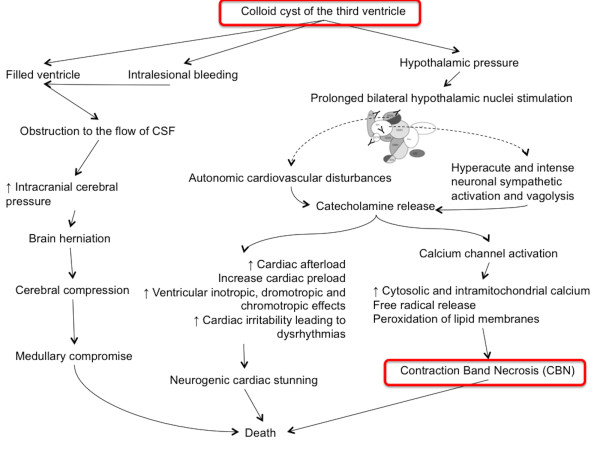
**Alternative mechanisms underlying the sudden death of patients with colloid cyst: hypothalamic structures which are involved in cardiovascular control are located close to the walls of the third ventricle which is most frequent anatomical site of colloid cyst.** This may suggest that reflex cardiac effects due to the compression of the hypothalamic cardiovascular regulatory centers by the cyst could explain the sudden death in patients harboring a colloid cysts when signs of hydrocephalus or brain herniation are lacking.

However, although the frequent post – mortem finding of ventricular enlargement and brain herniation in fatal case of patients with colloid cyst of the third ventricle had led most authors to postulate that hydrocephalus may play a major role in fatal cases [9,14,15], the accurate pathophysiologic lethal mechanism is not completely cleared. Moreover, it is interesting to note that neither the cyst size, nor the degree of ventricular dilatation appears to be a reliable predictor of outcome, as even small, asymptomatic cists may result in sudden death [7,16,17]. Such findings suggest an alternative mechanism underlying the sudden death of patients with colloid cyst. As the hypothalamic structures which are involved in neuroendocrine and autonomic regulation playing a key role in cardiovascular control [18,19] are located close to the walls of the third ventricle which is the most frequent anatomical site of colloid cyst, this may suggest that reflex cardiac effects due to the compression of the hypothalamic cardiovascular regulatory centers by the cyst explain the sudden death in patients harboring a colloid cysts when signs of hydrocephalus or brain herniation are lacking [3,7]. It is well known that stimulation of the hypothalamus can lead to autonomic cardiovascular disturbances [5,20]; bilateral prolonged stimulation of the hypothalamus produces cardiac alterations indistinguishable from that produced by catecholamine injections and stress, namely contraction band necrosis [5] (Figure 4).

In other words, “sympathotonic” prone individuals may have an “adrenergic crisis” any time a structural brain damage occurs, which explains the high variability among subjects of the same group. These pathological conditions may trigger a catecholamine myotoxicity and acting through free radical mediated lipid peroxidation with intramyocellular Ca2+ influx. In fact, we hypothesized that in the pathophysiology of CBN a role is played by the presence of reactive oxygen species (ROS) [21]. Exposure of normal myocardium to ROS-generating systems alters myocardial function through persistent cellular loss of K+, depletion of high-energy phosphates, elevated intracellular calcium concentration, loss of systolic force development, progressive diastolic tension, and depressed metabolic function [22,23]. Catecholamines may induce oxidative damage through reactive intermediates resulting from their auto-oxidation, irrespective of their interaction with adrenergic receptors, thus representing an important factor in the pathogenesis of catecholamines-induced cardiotoxicity [24]. In a previous paper we have described the effect of ROS on the catecholamine-mediated myocardial expressions of TNF-a (tumor necrosis factor-alpha), MCP-1 (monocyte chemotactic protein-1), interleukins IL6, IL8, IL10 and a significant apoptotic process randomly sparse in the damaged myocardium [25,26]. The rise of the cardio-inhibitory cytokines may be interpreted as the adaptive response of jeopardized myocardium with respect to the cardiac dysfunction resulting from catecholamines effects [25].

The presented histological investigation can contribute to elucidate the link between cardiac damage and colloid cyst of the third ventricle and to understand the exact pathophysiologic mechanism causing the sudden death of these patients when acute hydrocephalus and brain herniaton are not present. Coagulative myocytolysis may represent the histological hallmark of the sympathetic imbalance due to hypothalamic centers reflex stimulation independently from the size of the cyst and the amount of hydrocephalus. Considering this intriguing hypothesis surgical removal of colloid cysts of the third ventricle, even if small and asymptomatic, is mandatory [3,12,27].

## Consent

Written informed consent was obtained from the patient’s relatives for publication of this case report and any accompanying images.

## Competing interests

The authors declare that they have no competing interests.

## Authors’ contributions

ET and VF equally contributed to this article and conceived the study. ET and VF wrote the manuscript. SB, MN, IR made the pathological explorations. All authors read and approved the final manuscript.
